# Supplementary material for: Long COVID Prevalence and the Impact of the Third SARS-CoV-2 Vaccine Dose: A Cross-Sectional Analysis from the Third Follow-Up of the Borriana Cohort, Valencia, Spain (2020–2022)
Source: Vaccines (Basel). 2023 Oct 12;11(10):1590. doi: 10.3390/vaccines11101590 (PMC10611325; doi:10.3390/vaccines11101590)
Supplement: Supplementary file 1 [file vaccines-11-01590-s001.zip › vaccines-2613761-supplementary.pdf]

Supplementary materials

Laboratory methods

We first performed a serosurvey and an epidemiological questionnaire survey in June 2020, which has been described by Domenech-Montoliu and co-authors (1). A second serosurvey and questionnaire were conducted in October 2020. In summary, the serosurveys included determinations of anti-SARS-CoV-2 N-antibodies in June and October 2020 with a flow chart of the study, and ABO blood groups and vitamin D status were determined in October 2020. The serosurvey results were the subject of two previous publications and are not further discussed in the current manuscript (1, 2).

Supplement Cellular Immunity, methods

Two sets of 15-mer overlapping peptides (11-mer overlap) encompassing the ancestral Wuhan-Hu-1 SARS-CoV-2 Spike (S) glycoprotein (S1, 158 peptides and S2, 157 peptides) and in parallel with a pool of 315 (158+157) peptides derived from a peptide scan (15 mers with 11 aa overlap, including a few 13–17 mers) through Spike glycoprotein, containing mutations T19I, L24-, P25-, P26-, A27S, G142D, V213G, G339D, S371F, S373P, S375F, T376A, D405N, R408S, K417N, N440K, S477N, T478K, E484A, Q493R, Q498R, N501Y, Y505H, D614G, H655Y, N679K, P681H, N764K, D796Y, Q954H, N969K of SARS-CoV-2 (Severe Acute Respiratory Syndrome-Related Coronavirus 2)—(Lineage B.1.1.529 / BA.2 Omicron sub-variant), both at a concentration of 1 µg/ml per peptide (JPT Peptide Technologies GmbH; Berlin, Germany), in the presence of 1 µg/ml of costimulatory monoclonal antibodies (mAbs) to CD28 and CD49d, were used for 6 h stimulation (BD Biosciences). Appropriate positive (phytohemagglutinin) and negative controls were used. Samples mock-stimulated with phosphate-buffered saline (PBS)/dimethyl sulfoxide and costimulatory antibodies were run in parallel. Brefeldin A (10 µg/ml) was added for the last 4 h of incubation. Blood was then lysed (BD FACS lysing solution) and frozen at −80 °C until tested. On the day of testing, stimulated blood was thawed at 37 °C, washed, permeabilized (BD permeabilizing solution), and stained with a combination of labeled mAbs (anti-IFNγ-FITC, anti-CD4-APC-H7, anti-CD8-PerCP-Cy5.5, and anti-CD3-APC) for 1 h at room temperature (Table antibodies). Cells were then washed, resuspended in 200 µL of 1% paraformaldehyde in PBS, and analyzed within 2 h on an FACSCanto flow

cytometer (BD Biosciences Immunocytometry Systems, San Jose, CA). CD3+/CD8+ or CD3+/CD4+ events were gated and then analyzed for IFN-γ production. The data are expressed as the number of SARS-CoV-2-reactive IFN-γ-producing $CD4^+$ or $CD8^+$ T cells relative to the absolute number of $CD4^+$ and $CD8^+$ T cells, respectively, x100 (%). Any frequency value of SARS-CoV-2-reactive IFN-γ-producing $CD4^+$ or $CD8^+$ T cells after background subtraction was considered as a positive (detectable) result and used for analysis purposes (3).

References

1. Domenech-Montoliu S, Pac-Sa MR, Vidal-Utrillas P, Latorre-Poveda M, Del Rio-Gonzalez A, Ferrando-Rubert S, et al. "Mass gathering events and COVID-19 transmission in Borriana (Spain): A retrospective cohort study". PLoS One. 2021;16(8):e0256747.

2. Domènech-Montoliu S, Puig-Barberà J, Pac-Sa MR, Vidal-Utrillas P, Latorre-Poveda M, Del Rio-González A, et al. Persistence of Anti-SARS-CoV-2 Antibodies Six Months after Infection in an Outbreak with Five Hundred COVID-19 Cases in Borriana (Spain): A Prospective Cohort Study. Covid. 2021;1(1):71-82.

3. Tormo N, Gimenez E, Martinez-Navarro M, Albert E, Navalpotro D, Torres I, et al. Performance comparison of a flow cytometry immunoassay for intracellular cytokine staining and the QuantiFERON(R) SARS-CoV-2 test for detection and quantification of SARS-CoV-2-Spike-reactive-IFN-gamma-producing T cells after COVID-19 vaccination. Eur J Clin Microbiol Infect Dis. 2022;41(4):657-62.

Supplemental Table S1. Comparison of the characteristics of COVID-19 episodes by immunization status: analysis of primed individuals (with prior COVID-19 infection, having received one, or two doses) versus those who received a third booster dose.

	Third dose vs none or one or two previous doses			
	Primed	Boosted 3rd dose	Total	p-value
	154	319	473	
Covid Hx none, one episode and reinfection				
One episode	100 (64.9%)	253 (79.3%)	353 (74.6%)	<0.001
Reinfected	54 (35.1%)	66 (20.7%)	120 (25.4%)	
First episode				
Asymptomatic	27 (17.6%)	78 (24.5%)	105 (22.3%)	0.093
Symptomatic	126 (82.4%)	240 (75.5%)	366 (77.7%)	
1st Covid episode duration in days	9.91 (18.54)	8.08 (13.70)	8.66 (15.40)	0.245
1st episode lab test				
No	22 (14.3%)	43 (13.5%)	65 (13.7%)	0.811
Yes	132 (85.7%)	276 (86.5%)	408 (86.3%)	
Outpatinet consultation 1st episode				
No	70 (46.4%)	186 (58.3%)	256 (54.5%)	0.015
Yes	81 (53.6%)	133 (41.7%)	214 (45.5%)	
Admission				
No	145 (95.4%)	310 (97.5%)	455 (96.8%)	0.228
Yes	7 (4.6%)	8 (2.5%)	15 (3.2%)	
Second episode				
Asymptomatic	9 (16.7%)	14 (21.5%)	23 (19.3%)	0.503
Symptomatic	45 (83.3%)	51 (78.5%)	96 (80.7%)	
2n episode lab test				
No	3 (5.6%)	10 (15.2%)	13 (10.8%)	0.092
Yes	51 (94.4%)	56 (84.8%)	107 (89.2%)	
2nd COVID epsiode duration in days	4.40 (4.48)	4.22 (3.77)	4.30 (4.09)	0.814
Outpatinet consultation for COVID reinfection				
No	28 (51.9%)	35 (53.8%)	63 (52.9%)	0.828
Yes	26 (48.1%)	30 (46.2%)	56 (47.1%)	
Admission, reinfection episode				
No	54 (100.0%)	64 (98.5%)	118 (99.2%)	0.360
Yes	0 (0.0%)	1 (1.5%)	1 (0.8%)	
Sequelae				
No	97 (63.0%)	231 (72.4%)	328 (69.3%)	0.037
Yes	57 (37.0%)	88 (27.6%)	145 (30.7%)	
Fully recovered after covid				
No	42 (27.3%)	63 (19.7%)	105 (22.2%)	0.065
Yes	112 (72.7%)	256 (80.3%)	368 (77.8%)	
Perceived Current Health Status				
Poor	5 (3.3%)	6 (1.9%)	11 (2.3%)	0.479
Fair	27 (17.6%)	47 (14.8%)	74 (15.7%)	
Good	90 (58.8%)	208 (65.6%)	298 (63.4%)	
Excellent	31 (20.3%)	56 (17.7%)	87 (18.5%)	
As healthy as before				
No	44 (28.6%)	80 (25.1%)	124 (26.2%)	0.418
Yes	110 (71.4%)	239 (74.9%)	349 (73.8%)	
Scoring GAD-7 Anxiety Severity				
0-4; minimal anxiety	95 (61.7%)	230 (72.1%)	325 (68.7%)	0.038
5-9; mild anxiety	31 (20.1%)	57 (17.9%)	88 (18.6%)	
10-14; moderate anxiety	20 (13.0%)	19 (6.0%)	39 (8.2%)	
15-21; severe anxiety	8 (5.2%)	13 (4.1%)	21 (4.4%)	
Axiety, mild top severe GAD-7 score				
No	95 (61.7%)	230 (72.1%)	325 (68.7%)	0.022
Yes	59 (38.3%)	89 (27.9%)	148 (31.3%)	
Depression severity measure				
No or minimal	116 (75.3%)	249 (78.1%)	365 (77.2%)	0.339
Mild	27 (17.5%)	44 (13.8%)	71 (15.0%)	
Moderate	8 (5.2%)	14 (4.4%)	22 (4.7%)	
Moderate to Severe	1 (0.6%)	10 (3.1%)	11 (2.3%)	
Severe	2 (1.3%)	2 (0.6%)	4 (0.8%)	
Depression, PHQ-9 score				
No	116 (75.3%)	249 (78.1%)	365 (77.2%)	0.507
Yes	38 (24.7%)	70 (21.9%)	108 (22.8%)	

Supplementary Table S2. Participant characteristics associated with immunization, comparing between those primed (with prior COVID-19 infection and having received one, or two doses) and those who received a third booster dose.

	Third dose vs one or two previous doses				
	Primed	Boosted 3rd dose	Total	p-value	SMD
	142	319	461		
Age in years	38.96 (12.19)	43.35 (12.98)	42.00 (12.89)	<0.001	-0.35
Age in groups in years					
18-24	25 (17.6%)	42 (13.2%)	67 (14.5%)	0.009	0.32
25-49	85 (59.9%)	159 (49.8%)	244 (52.9%)		
50-64	32 (22.5%)	118 (37.0%)	150 (32.5%)		
Male/Female					
Male	51 (35.9%)	116 (36.4%)	167 (36.2%)	0.926	<0.2
Female	91 (64.1%)	203 (63.6%)	294 (63.8%)		
Blood group					
0	56 (39.7%)	140 (44.0%)	196 (42.7%)	0.842	<0.2
A	66 (46.8%)	138 (43.4%)	204 (44.4%)		
B	14 (9.9%)	28 (8.8%)	42 (9.2%)		
AB	5 (3.5%)	12 (3.8%)	17 (3.7%)		
Occupational class					
High/Middle/Qualif	81 (58.3%)	203 (66.1%)	284 (63.7%)	0.149	0.24
Manual	44 (31.7%)	70 (22.8%)	114 (25.6%)		
Low	6 (4.3%)	21 (6.8%)	27 (6.1%)		
Other	8 (5.8%)	13 (4.2%)	21 (4.7%)		
Educational level					
No schooling compl	3 (2.1%)	2 (0.6%)	5 (1.1%)	0.477	<0.2
Elementary School	15 (10.6%)	39 (12.5%)	54 (11.9%)		
High School	59 (41.8%)	122 (39.2%)	181 (40.0%)		
University	64 (45.4%)	148 (47.6%)	212 (46.9%)		
Household members					
One or two	26 (18.4%)	69 (21.9%)	95 (20.8%)	0.080	0.26
Three	38 (27.0%)	108 (34.3%)	146 (32.0%)		
Four	70 (49.6%)	116 (36.8%)	186 (40.8%)		
More than four	7 (5.0%)	22 (7.0%)	29 (6.4%)		
Level of social activity					
Minimal	36 (25.4%)	87 (27.4%)	123 (26.7%)	0.106	0.21
Active	40 (28.2%)	115 (36.2%)	155 (33.7%)		
High	66 (46.5%)	116 (36.5%)	182 (39.6%)		
Activity in contact with people					
No	25 (17.7%)	70 (22.1%)	95 (20.7%)	0.289	<0.2
Yes	116 (82.3%)	247 (77.9%)	363 (79.3%)		
Regular physical activity					
No	65 (45.8%)	142 (44.5%)	207 (44.9%)	0.802	<0.2
Yes	77 (54.2%)	177 (55.5%)	254 (55.1%)		
Smoker					
No	81 (57.9%)	189 (59.6%)	270 (59.1%)	0.811	<0.2
Ex	27 (19.3%)	64 (20.2%)	91 (19.9%)		
Current	32 (22.9%)	64 (20.2%)	96 (21.0%)		
Alcohol consumption					
None	41 (28.9%)	78 (24.5%)	119 (25.8%)	0.317	<0.2
Light to moderate	101 (71.1%)	241 (75.5%)	342 (74.2%)		
Underlying disorders					
No	103 (73.0%)	210 (66.7%)	313 (68.6%)	0.175	<0.2
Yes	38 (27.0%)	105 (33.3%)	143 (31.4%)		
bmi	25.75 (5.25)	26.63 (4.98)	26.36 (5.07)	0.085	<0.2
Vit D blood levels					
<30 UI	78 (54.9%)	198 (62.1%)	276 (59.9%)	0.149	<0.2
30 UI and over	64 (45.1%)	121 (37.9%)	185 (40.1%)		
Covid Hx none, one episode and reinfection					
One episode	97 (68.3%)	253 (79.3%)	350 (75.9%)	0.011	0.25
Reinfected	45 (31.7%)	66 (20.7%)	111 (24.1%)		
Long COVID					
No	90 (63.4%)	243 (76.2%)	333 (72.2%)	0.005	0.28
Yes	52 (36.6%)	76 (23.8%)	128 (27.8%)		
COVID cases by Predominant VOC circulation in Spain					
Ancestral	94 (66.7%)	211 (66.4%)	305 (66.4%)	0.014	0.30
Alpha	11 (7.8%)	6 (1.9%)	17 (3.7%)		
Delta	1 (0.7%)	2 (0.6%)	3 (0.7%)		
Omicron	35 (24.8%)	99 (31.1%)	134 (29.2%)		
log CD4+ Wuhan	-6.58 (1.15)	-6.62 (1.10)	-6.61 (1.11)	0.845	
log CD8+ Wuhan	-6.33 (1.55)	-6.07 (1.40)	-6.16 (1.45)	0.379	
log CD4+ BA.2	-6.43 (1.13)	-6.58 (1.17)	-6.53 (1.15)	0.504	
log CD8+ BA.2	-6.41 (1.38)	-6.23 (1.40)	-6.28 (1.39)	0.528	
Anti-S AU	1732.75 (1421.24)	2216.07 (1627.33)	2067.20 (1581.06)	0.002	
Anti-N AU	2.17 (2.76)	2.47 (2.82)	2.38 (2.81)	0.301	

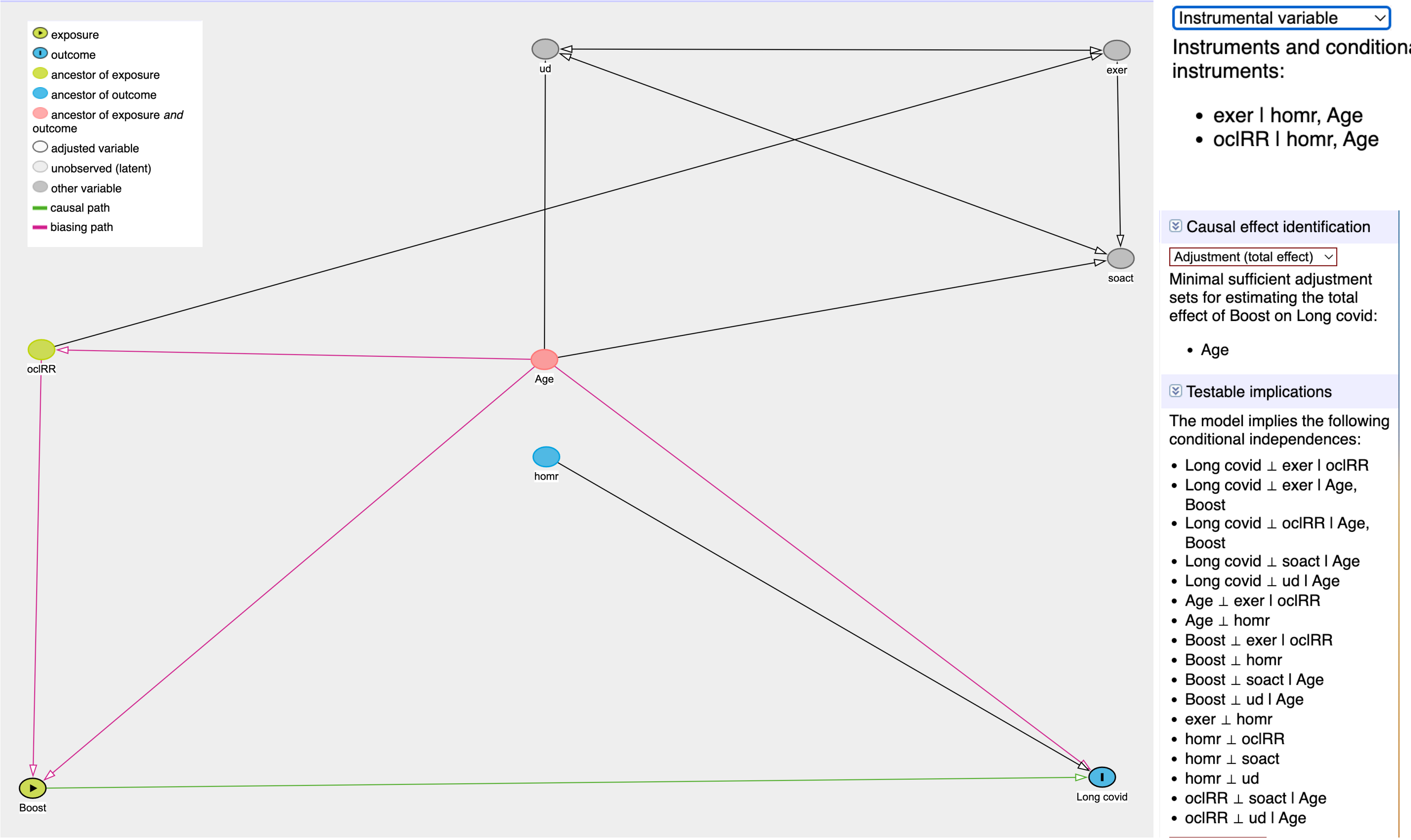

exer: regular physical activity. homr: Number of household members. oclRR: occupational class. ud: underlying chronic illnesses. soact: socila activity intensity. Boost: booster dose.

Supplementary Figure S1: DAG analysis.
